# The Moral Side of Dentistry (Third Paper)

**Published:** 1902-02-15

**Authors:** Geo. W. Clapp

**Affiliations:** Cygnet, Ohio


THE
### DENTAL REGISTER.

Vol. LVI. February 15, 1902. No. 2.

### THE MORAL SIDE OF DENTISTRY.

(*THIRD PAPER.*)

BY GEO. W. CLAPP, D.D.S., CYGNET, OHIO.

At the very beginning of this article I want to place some words of thanks to those who have made the gathering of materials for it possible. That men of world-wide fame and great enterprises should pause to assist a young stranger in a self-assumed task is worthy of note, and I take this opportunity of expressing my gratitude.

It would be but poor return for such help to write in an unkind criticism of these men or the institutions over which they preside. However near any statements in this article may come to any of them, I hope they will accept them as written, in a spirit of kindness and helpfulness.

The materials for this paper were gathered in the following manner: Several colleges which were thought to represent the best spirit and practice of dental education were selected, and a copy of the following letter was addressed to the dean or secretary of each faculty.

The list did not include every prominent college, and some school not addressed may have in practical operation much better plans than those quoted. While this is possible, it is not probable ; if it were so it would not controvert the applications herein made, since all that is sought is the elucidation of principles. The schools replying to the letter represent a student body nearly sixteen hundred strong. In three years most of these students will be dentists. Every three years these schools will graduate at least this number, Thus, any thing which affects these schools is of great importance to the profession. The nine questions in the letter are intended to cover two main points, wholly from the side of morals. They are: First, the pains taken to investigate the student's character previous to admitting him to the school ; and second, the pains taken by the faculties to acquaint themselves with the student's moral fitness previous to graduation.

The letter was as follows:

"*Dear Doctor:* I am preparing for THE DENTAL REGISTER a series of articles on 'The Moral Side of Dentistry. For the purpose of securing necessary information, and to avoid misrepresentation, I am addressing to our leading faculties the following list of questions. Will you kindly answer them for your school? Your answers will be treated as confidential.

"1. What is your present enrollment?

"2. Do you make any systematic effort to investigate the personal character of each student? If so, what?

"3. To this end do you address inquiries to those who knew the student in the past? If so, to how many persons?

"4. If their replies are unfavorable, what course do you pursue?

"5. Do you suspend or expel a student for known affliction with syphilis, gonorrhoea, alcoholism, licentiousness, or other moral aberration?

"6. How many have you expelled for such cause during the past three years?

"7. What effort do you make, while the student is under your charge, to determine whether by character and habit of life he is fit to become a professional man?

"8. If you have reason to believe that his future career will not be creditable to the profession, what course do you pursue?

"9. Will you please mail me copies of such questions as you send to students, or others, preparatory to their entering your school?"

Questions 2, 3 and 9 deal with investigating the prospective student's character before his admission, with a view of selecting as students such as are morally qualified to practice with honor. I wish it were within my power to impress upon every dental faculty the importance of such selection. There are some duties for which the selection of men need not be especially careful, but the duties committed to the professional man are not among them. The professional man's duties are among the most important in life, and it often makes all the difference between life and death whether or not they are well done. Of none is this truer than of the tasks committed to medicine and its allies, and the qualifications for their proper discharge should be keenly scrutinized in each applicant.

In view of the trouble which all good schools take to select those who are mentally qualified, it is both surprising and discreditable to learn that none of the schools addressed makes adequate efforts to select as students only those who are morally fit. This statement may arouse contradiction, but personal knowledge of the student body of several leading schools bears it out. Such remissness is the more deplorable, because, from even the most material standpoint, the immoral man is not equal to the moral, other things being equal; nor is the brain which has been weakened by dissipation and is now fired by stimulants equal to the same brain acting serenely upon the foundation and with the force furnished by observance of the moral laws. The adult life gathers direction and momentum out of its past, and the ability to meet crises or to give each daily duty patient and skillful attention is generated by nothing else so well as by habits of moral rectitude. There are some surgeons of whom it is said that they never operate so well as when stimulated by liquor. Under the requirements of modern surgery, however, such men are becoming more and more rare, and when their lives are fully known they are not such as appeal to the man seeking true success. If in the hour of stimulation it can render a great service, it is permissible that a brain weakened by excesses should be stimulated to the highest power of which it is capable; but it is manifestly better that a man should rise gradually to his maximum through the momentum of a wisely ordered life and maintain that level through the years of his greatest efficiency.

The most that any of these schools require of a prospective student is that he shall give names of two persons to whom, in the words of the secretary, "it is the intention to write." It is not stated how often the intention is carried out. The college with which this dental school is associated is one of the best in the world. Its name is known and its praises are sung wherever men seek high education. Its diploma says for a man all that any diploma says. The dental faculty desires to do its best. It is no light task to pass their intellectual requirements for entrance and no proposition for lowering them would be seriously considered. Yet the only moral requirements for entrance is that the matriculate shall give the names of two persons "to whom it is the intention to write." The practical value of the intention is shown when the dean writes in answer to the question whether they expel for known affliction with gonorrhea, syphilis, alcoholism, licentiousness, etc. "We never have had such a student." Either this school stands alone among dental schools for unassailable morality or the dean is woefully ignorant of the personal characters of the men under him; and the chances are one thousand to one in favor of the latter.

Other schools require the applicant to submit one or more letters testifying to his good personal character. These letters are usually accepted without investigation. They may testify to the excellence of the applicant in any trivial point and remain silent regarding the great characteristics. This point will be more fully covered in another paragraph.

A still more flimsy reliance is that put by some faculties in their power "to size a man up" morally, when he presents himself for admission. A bad character does not always mar the face at the age of most matriculates, nor can the phrenology of the skull be relied upon as infallible. The writer is acquainted with some of the faculty and several graduates at one school where they depend a great deal on "sizing a man up" and "require him to present a letter from a dentist, medical man or preacher who knows him well." A member of the faculty not engaged in "sizing up" stated that at one time there were nine syphilitics in the senior class whom the eagle eye of the "sizer-up" had not detected. A reputable graduate told me that he knew of nearly a dozen cases of gonorrhea in his class and that several of his classmates had been in jail during that term as the result of drunken carousals. "Sizing up" indeed!

Since so much fault is being found with the methods of investigation now in use, it is but fair to point out the weaknesses which insure their failure. These weaknesses fall under two heads. First, they permit the student, with the aid of a couple of friends, to fulfill the requirements however bad his character may be. There are men, including some dentists, whose moral codes are so lax as to permit them to write letters endorsing anybody or anything. Second, since the required letter is not in reply to specific inquiries, it need not be specific in its endorsement. There are few young men but have some good qualities and since the writer is at liberty to commend any good traits and remain silent concerning any of which he does not approve, almost any friend can write a letter commending the would-be student. For instance: A desires B, a dentist, to write an endorsement. A is a young man of average ability, of pleasing manners and a friendly disposition; he wears good clothes, dances well and is a "good fellow" generally. He uses liquor and is occasionally the worse for it. He associates with fast women and suffers the usual consequences. He is also a more frequent borrower than payer. B knows this, but B is a "good fellow" himself and writes as follows:

"I have known Mr. A intimately for several years. In my opinion his abilities fit him to become an acceptable student of dentistry. His social education and pleasing address qualify him to appeal to any class of people among whom he may desire to practice."

A little sifting will show that this letter does not make a single statement of any moral consequence. B has avoided saying anything. Yet such a letter would be accepted as a credential of a good moral character by most schools. If the writer's evasions and mental reservations were expressed in each letter, many letters would be very different documents.

The results of such carelessness in selection would be far less serious if the student were subjected to close and intelligent scrutiny and discipline during his course, but he is not. Replies to the circular letter show that every school relies upon the same means, so far as any are used, to supervise the students. The secretaries write; "The professors and demonstrators are in close touch with the students during their time here." Maybe the secretaries think this the fact, but it isn't. Not one professor in ten is in anything like close touch with the students. It is not possible for them to be, save in very small schools. Take some actual cases. Prof. L. lectures to medical and dental students five days a week during the school year. About two hundred and fifty men are bunched compactly before him for forty or fifty minutes. He learns to know the faces immediately before him and the half dozen strongest men in his quiz section. He says more is impossible. Is that being in close touch with the men? Dr. C. has a down-town office where he is busy at remunerative prices. He lectures to the students three days a week at ten o'clock. He works at the office until the last minute, hurries to college, faces fifty or one hundred men, for fifty minutes and is back at the office in time to work an hour before lunch. How close is his touch with the men? How much time for attention and thought concerning them does he have? He doesn't know them when he sees them ; how much *can* he know about them morally.

With the demonstrators it is different. They are usually less removed by age and standing from the students. They are more nearly at one with the men and their knowledge of the students they supervise is usually adequate, but this knowledge so far as it concerns private immoralities on the student's part, rarely reaches the faculty in such shape as to cause official action. If the student will keep up the work for which that demonstrator is responsible he need fear no consequences from any private debaucheries in which he may indulge.

The reason why such knowledge fails to become action has been given in previous papers. It is because the profession as a body has not required anything of the private morals of its members, and therefore can not bring pressure to bear on the schools. If an adequate sense of responsibility could be inculcated in each faculty and each member could be made to feel that the private morals of each student is a legitimate subject of inquiry and executive action—yea, that it is a subject on which a faculty *must* be satisfied before any diploma could be granted—the quietest, the greatest and the most beneficient revolution possible to dentistry would be complete.

The plan proposed below is neither new nor original, nor does it require any radical departure from the plans now nominally (and only nominally) in force at many schools. Neither is it supposed that the plan is perfect. It is a suggestion based on the plans of those companies who insure the honesty of employees wholly upon information obtained in this way. They find it sufficiently effective to insure men for large sums upon it, and make such insurance pay.

The plan is based on the assumption that if a man's past has been filled with debauchery he is probably bad in the present, and will bear the closest watching. If his past has been reasonably clean, his present will probably be so. In either case the plan permits the faculty to know what manner of man each student is, and to refuse undesirable matriculations.

Any plan which can be proposed is open to two insuperable objections. First, the faculty is unwilling to take the trouble to procure information. Second, that the faculty does not care what the morals of its graduates are.

There are, unfortunately, many good teachers whose perception of moral responsibility is much less keen than their perception of mental responsibility. They do not question their right to enquire into a student's intellectual past, or to require of him a certain grade of work. Neither would they hesitate to enfore certain physical restrictions. They would suspend from class any case of infectious disease. But so little alive to moral responsibilities are they that there daily come before them men of whose past and present they know nothing and who, for all they know, may be foci of infection deadly to morality and even decency. If these moral lepers can only answer the class quiz or examination satisfactorily such teachers do not hesitate to indorse them to the world. Yet they would heartily resent a charge of moral malpractice. It may also be that some faculties do not care what manner of men they matriculate, so long as each man pays his fees promptly. Other things being equal they prefer good men but men and fees are what they want.

The plan proposed for overcoming this evident weakness presents its strongest points just where the present plan is weakest. It requires replies to similar questions from a number of persons sufficient to prevent any collusion between the student and the referees. The questions are searching and specific and cover the points on which the faculty should be informed. They prevent a meaningless general indorsement.

The plan first requires the student to fill out a form which furnishes the names of several people, to any desired number to whom inquiries may be addressed, such as the following :

FORM FOR STUDENT.

Full name. . . . . . . . . . . . . . . . . . . . . . . . . . . . . . . . Age. . . . . . . . . .
Born at. . . . . . . . . . . . . . . . . . . . . . . . . . . . Date . . . . . . . . . . . . . . .
Single. . . . . . . . . . . Married. . . . . . . . . . Widower. . . . . . . . . .
Residence address . . . . . . . . . . . . . . . . . . . . . . . . . . . . . . . . . . .
Business address. . . . . . . . . . . . . . . . . . . . . . . . . . . . . . . . . . . . .
Where have you previously resided? Give dates. . . . . . . . .
What schools have you attended? Give dates . . . . . . . . . . .
Name your two last teachers and the principal, and give the city or town in which they live. . . . . . . . . . . . . . . . . .
If you have been in business, give names and addresses of employers and dates in their service. . . . . . . . . . . . . . . . . .
Were you discharged from any of the above employments? If so, from which, and for what cause?. . . . . . . . . . . . . . . .
Are you a member of any church? If so, give name and address of pastor. . . . . . . . . . . . . . . . . . . . . . . . . . . . . . . . .
Do you use intoxicating liquors?. . . . . . . . . . . . . . . . . . . . . . . . .
Do you visit houses of ill fame? . . . . . . . . . . . . . . . . . . . . . . . .
Give names and addresses of one or two dentists who know you well but to whom you are no relation . . . . . . . . . . . . .
Give name and address of your family physician. . . . . . . . . .
Give names and addresses of two friends who know you well but are not related to you . . . . . . . . . . . . . . . . . . . . . . .

The following form should then be addressed to the teachers, employers, friends and dentists mentioned above :

Mr. . . . . . . . . . . . . . . . . . .has applied for studentship in this

College, and has referred us to you that we may inform ourselves concerning his personal character. Will you kindly answer the following questions and mail to us as early as is convenient? Your answers will be considered strictly confidential and will involve no risk to you.

1. How long have you known the applicant?............
2. Are you related to him in any way?...................
3. Do you believe him to be trustworthy? If not, in what particulars?.........................................
4. Have you ever known of his using intoxicating liquors in moderation?...... In excess?...... If so, how long ago?.........................................
5. Have you ever known of his associating with people of immoral characters, or frequenting houses of ill fame? If so, how long since?.............................
6. Do you know of anything about him which would unfit him to make such a dentist as you would wish to employ?.........................................

The questions to the family physician might well be more specific.

1. How long have you known family of applicant?.......
2. Are you related to him in any way?...................
3. Is either parent afflicted with any disease which would be likely to injure applicant?.......................
4. Has applicant any hereditary taint?...................
5. So far as you know, is he of good character?..........
6. Do you know or have you heard of his ever having syphilis, gonorrhea, alcoholism or tuberculosis?
7. Do you know or have you heard of his associating with people of immoral character, or frequenting houses of ill fame?..... ...................................
8. Do you know of any reason why he might not become a creditable professional man?.......................

We close these articles with an expression of hope that they may start the thought in the minds of some who are in positions where they can effectively inaugurate some such reform as has been outlined.

